# Supplementary material for: DEC1 regulates human β cell functional maturation and circadian rhythm
Source: Cell Rep. Author manuscript; Available in PMC 2026 Feb 13. (PMC12904728; doi:10.1016/j.celrep.2025.116666)
Supplement: 1 [file NIHMS2132754-supplement-1.pdf]

Cell Reports, Volume *44*

Supplemental information

DEC1 regulates human β cell functional maturation and circadian rhythm

Sam Preza, Bliss Zheng, Zihan Gao, Akshaya Biju, Mai Liu, Zhihui Cheng, Matthew Choi, and Juan R. Alvarez-Dominguez

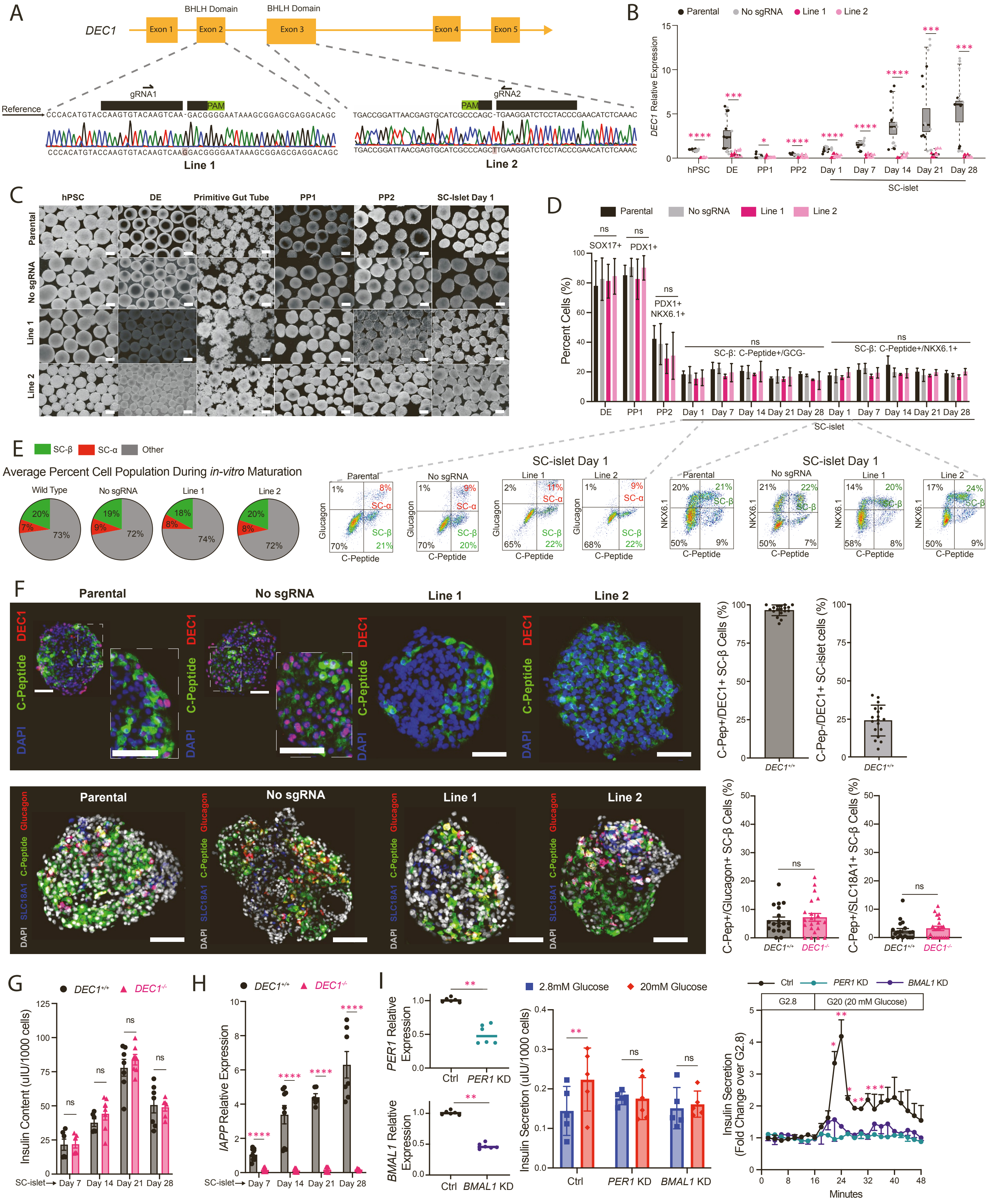
A
DEC1
Exon 1
Exon 2
Exon 3
Exon 4
Exon 5
BHLH Domain
BHLH Domain
gRNA1
PAM
Reference
CCCACATGTACCAAGTGTACAAGTCAA-GACGGGGAATAAAGCGGAGCGAGGACAGC
CCCACATGTACCAAGTGTACAAGTCAAGGACGGGGAATAAAGCGGAGCGAGGACAGC
Line 1
PAM
gRNA2
TGACCGGATTAACGAGTGCATCGCCCAGC-TGAAGGATCTCCTACCCGAACATCTCAAC
TGACCGGATTAACGAGTGCATCGCCCAGCTTGAAGGATCTCCTACCCGAACATCTCAAC
Line 2
B
Parental
No sgRNA
Line 1
Line 2
DEC1 Relative Expression
hPSC
DE
PP1
PP2
Day 1
Day 7
Day 14
Day 21
Day 28
SC-islet
C
hPSC
DE
Primitive Gut Tube
PP1
PP2
SC-Islet Day 1
Parental
No sgRNA
Line 1
Line 2
D
Parental
No sgRNA
Line 1
Line 2
Percent Cells (%)
SOX17+
PDX1+
PDX1+ NKX6.1+
SC-β: C-Peptide+/GCG-
SC-β: C-Peptide+/NKX6.1+
ns
DE
PP1
PP2
Day 1
Day 7
Day 14
Day 21
Day 28
SC-islet
E
SC-β
SC-α
Other
Average Percent Cell Population During in-vitro Maturation
Wild Type
No sgRNA
Line 1
Line 2
20%
7%
73%
19%
9%
72%
18%
8%
74%
20%
8%
72%
SC-islet Day 1
Parental
No sgRNA
Line 1
Line 2
Glucagon
C-Peptide
SC-α
SC-β
1%
8%
70%
21%
1%
9%
70%
20%
2%
11%
65%
22%
1%
9%
68%
22%
SC-islet Day 1
NKX6.1
20%
21%
50%
9%
21%
22%
50%
7%
14%
20%
58%
8%
17%
24%
50%
9%
F
Parental
No sgRNA
Line 1
Line 2
DEC1
C-Peptide
DAPI
C-Pep+/DEC1+ SC-β Cells (%)
C-Pep-/DEC1+ SC-islet cells (%)
DEC1+/+
Glucagon
SLC18A1
C-Pep+/Glucagon+ SC-β Cells (%)
C-Pep+/SLC18A1+ SC-β Cells (%)
DEC1+/+
DEC1-/-
G
DEC1+/+
DEC1-/-
Insulin Content (uIU/1000 cells)
SC-islet→
Day 7
Day 14
Day 21
Day 28
H
IAPP Relative Expression
I
PER1 Relative Expression
Ctrl
PER1 KD
BMAL1 Relative Expression
BMAL1 KD
2.8mM Glucose
20mM Glucose
Insulin Secretion (uIU/1000 cells)
Ctrl
PER1 KD
BMAL1 KD
Ctrl
PER1 KD
BMAL1 KD
G2.8
G20 (20 mM Glucose)
Insulin Secretion (Fold Change over G2.8)
Minutes

Figure S1. DEC1 has no effect on SC-islet differentiation, composition, or insulin production. Related to Figure 1.

(A) Strategy to generate *DEC1*$^{-/-}$ hPSC lines for subsequent differentiation. Shown are CRISPR/Cas9 sgRNAs used to create two *DEC1*$^{-/-}$ hPSC lines, along with sequencing results indicating homozygous knockout due to frameshift-causing base-pair insertions.
(B) *DEC1* expression is abrogated throughout *in vitro* differentiation and maturation of SC-islets generated from *DEC1*$^{-/-}$ hPSC lines. Data from N = 4 differentiations with n = 2-3 technical replicate measurements.
(C) 3D SC-islet organoids form normally from *DEC1*$^{-/-}$ hPSC lines. Data are brightfield images of 3D organoids throughout differentiation. Scale bar is 100um.
(D) SC-islets differentiate normally from *DEC1*-ablated hPSC lines. Data quantify the indicated cell populations by flow cytometry for the indicated differentiation markers (N = 5 differentiations, n ≥ 2000 cells each). Representative flow cytometry data are shown in S1E.
(E) SC-islet composition is not affected by DEC1 loss. Data is average of SC-β (C-Peptide+/Glucagon-), and SC-α (Glucagon+) cells across day 7, 14, 21 and 28 for the indicated lines (N = 5 differentiations, n ≥ 2000 cells each). Representative flow cytometry data for differentiation marker quantification are shown to the right.
(F) (Top) C-Peptide and DEC1 immunostaining of day 14 *DEC1*$^{+/+}$ and *DEC1*$^{-/-}$ SC-islets. Scale bar is 50um. Percent of C-peptide positive cells that are DEC positive, and percent of DEC1 positive cells that are C-peptide negative from N=5 *DEC1*$^{+/+}$ differentiations (n = 3-4 SC-islet organoids each) (right). (Bottom) Percent of polyhormonal cells is not affected by DEC1 loss. C-Peptide, Glucagon, and SLC18A1 immunostaining of day 14 *DEC1*$^{+/+}$ and *DEC1*$^{-/-}$ from N=5 *DEC1*$^{+/+}$ differentiations (n = 3-4 SC-islet organoids each). Percent of C-peptide positive cells that are glucagon positive, and percent of C-peptide positive cells that are SLC18A1 positive (right).
(G) *DEC1*-ablated SC-islets produce insulin normally. Data is insulin content from day 7, 14, 21 and 28 *DEC1*$^{+/+}$ and *DEC1*$^{-/-}$ SC-islets (N=3 differentiation, n = 30-80 SC-islets each).

(H) *IAPP* expression is significantly reduced in *DEC1*$^{-/-}$ SC-islets from week 1 through week 4 of extended *in vitro* culture. Data from N = 3 differentiation with n = 2-3 technical replicate measurements, normalized to day 7 SC-islets.

(I) *BMAL1* and *PER1* siRNA knockdown (KD) in day 14 SC-islets show diminished glucose stimulated insulin secretion (GSIS) response. (Left) siRNAs successfully deplete *PER1* and *BMAL1* mRNA compared to siRNA control (Ctrl) conditions (N=3 differentiations, n=2 technical replicates each). (Middle) Diminished insulin response in static sequential incubations of 2.8mM glucose to 20mM glucose observed in day 14 *BMAL1* and *PER1* KD SC-islets (N=3 differentiations, n=50-100 islets each, assayed at least in duplicate). GSIS dynamics reveal day 14 *BMAL1* and *PER1* KD SC-islets sustain a low peak (first phase) insulin secretion under glucose stimulation (20mM) and dampened second phase secretion, consistent with the immature phenotype of *DEC1*$^{-/-}$ SC-islets. Data from day 14 *DEC1*$^{+/+}$ (N=3 differentiation, n = 50-100 islets) perifused with the indicated substrates at least in duplicate, normalized to the mean of the first incubation.

Data are mean ± SEM. *p <0.05, **p <1e-2, ***p <1e-3, ****p <1e-4, Wilcoxon test [(B), and (H)], and unpaired Welch's t test [(D), (E) against Parental line; (F), (G), (H) and (I)].

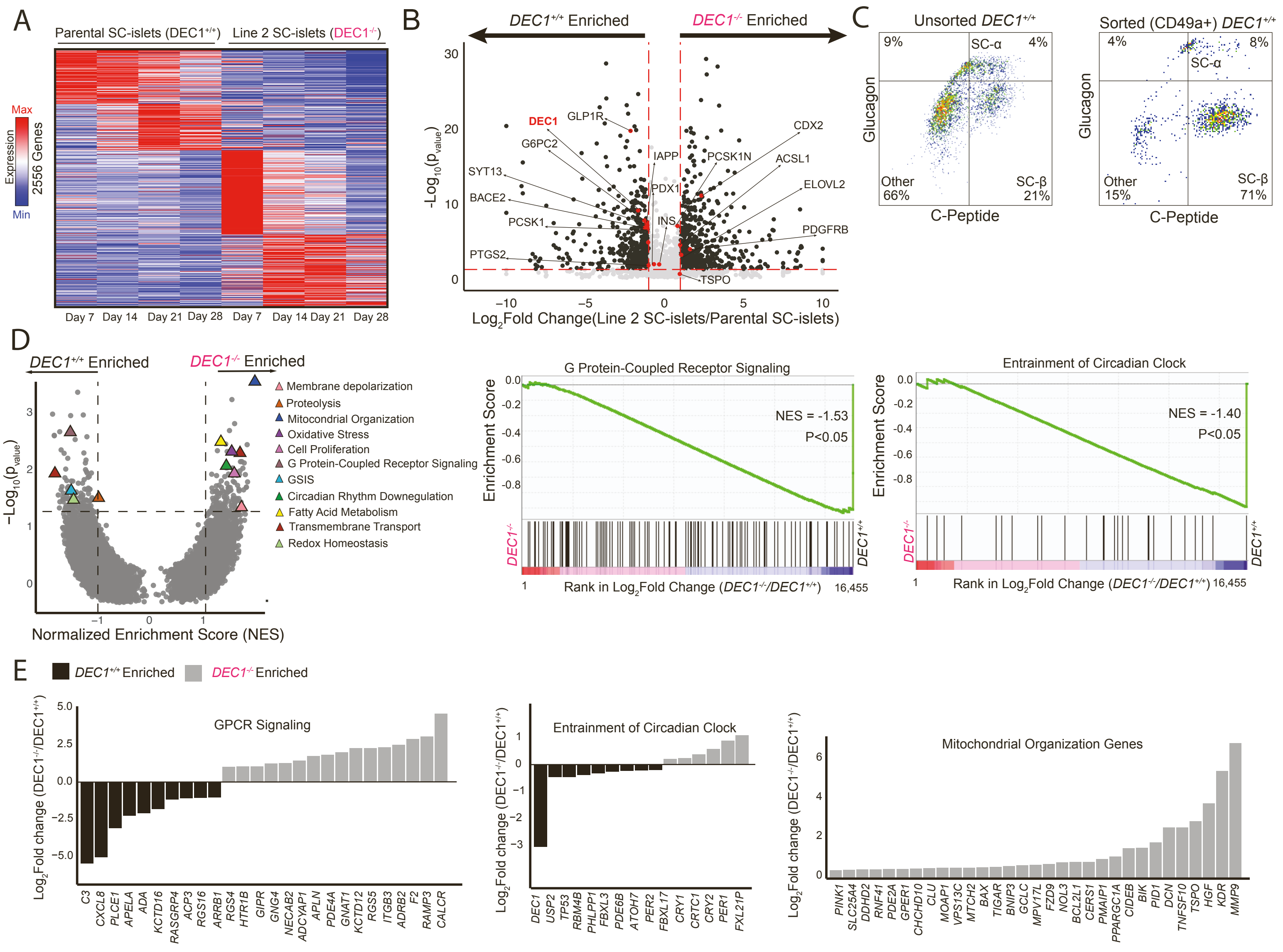

A
Parental SC-islets (DEC1+/+)
Line 2 SC-islets (DEC1-/-)
Max
Expression
2556 Genes
Min
Day 7 Day 14 Day 21 Day 28 Day 7 Day 14 Day 21 Day 28
B
DEC1+/+ Enriched
DEC1-/- Enriched
-Log10(pvalue)
DEC1
GLP1R
G6PC2
SYT13
BACE2
PCSK1
PTGS2
IAPP
PDX1
INS
PCSK1N
CDX2
ACSL1
ELOVL2
PDGFRB
TSPO
Log2Fold Change(Line 2 SC-islets/Parental SC-islets)
C
Unsorted DEC1+/+
Sorted (CD49a+) DEC1+/+
Glucagon
C-Peptide
9%
SC-α
4%
Other
66%
SC-β
21%
4%
8%
SC-α
Other
15%
SC-β
71%
D
DEC1+/+ Enriched
DEC1-/- Enriched
−Log10(pvalue)
Normalized Enrichment Score (NES)
Membrane depolarization
Proteolysis
Mitocondrial Organization
Oxidative Stress
Cell Proliferation
G Protein-Coupled Receptor Signaling
GSIS
Circadian Rhythm Downegulation
Fatty Acid Metabolism
Transmembrane Transport
Redox Homeostasis
G Protein-Coupled Receptor Signaling
Enrichment Score
NES = -1.53
P<0.05
DEC1-/-
DEC1+/+
Rank in Log2Fold Change (DEC1-/-/DEC1+/+)
16,455
Entrainment of Circadian Clock
NES = -1.40
P<0.05
E
DEC1+/+ Enriched
DEC1-/- Enriched
Log2Fold change (DEC1-/-/DEC1+/+)
GPCR Signaling
C3 CXCL8 PLCE1 APELA ADA KCTD16 RASGRP4 ACP3 RGS16 ARRB1 RGS4 HTR1B GIPR GNG4 NECAB2 ADCYAP1 APLN PDE4A GNAT1 KCTD12 RGS5 ITGB3 ADRB2 F2 RAMP3 CALCR
Entrainment of Circadian Clock
DEC1 USP2 TP53 RBM4B PHLPP1 FBXL3 PDE6B ATOH7 PER2 FBXL17 CRY1 CRTC1 CRY2 PER1 FXL21P
Mitochondrial Organization Genes
PINK1 SLC25A4 DDHD2 RNF41 PDE2A GPER1 CHCHD10 CLU MOAP1 VPS13C MTCH2 BAX TIGAR BNIP3 GCLC MPV17L FZD9 NOL3 BCL2L1 CERS1 PMAIP1 PPARGC1A CIDEB BIK PID1 DCN TNFSF10 TSPO HGF KDR MMP9

Figure S2. Additional gene set enrichment analysis of *DEC1*$^{-/-}$ SC-β cells. Related to Figure 2.

(A) *DEC1* ablation in SC-islet organoids results in 2,556 differentially expressed genes. Heatmap shows differentially expressed genes ($p < 0.05$, DEseq test) in Parental and *DEC1*$^{-/-}$ Line 2 SC-islets at days 7, 14, 21, and 28 of *in vitro* maturation.

(B) SC-β maturity-linked genes are depleted in *DEC1*$^{-/-}$ SC-islets. Volcano plot shows all differentially expressed genes at day 21 of SC-islet *in-vitro* maturation. Genes with at least a 2-fold change and p-value less than 0.05 are in black. Genes of interest are highlighted.

(C) Flow cytometric sorting of CD49a-positive cells enables RNA-sequencing of highly enriched SC-β populations from day 14 SC-islets. $n \geq 2000$ cells.

(D) *DEC1* ablation results in dysregulation of important β-cell processes in SC-β cells (p-value < 0.05 and absolute NES value > 1) (left). Gene set enrichment analysis shows genes ranked by fold change in day 14 *DEC1*$^{-/-}$ vs. *DEC1*$^{+/+}$ SC-β cells (middle and right). NES, normalized enrichment score.

(E) *DEC1* ablation upregulates genes modulating mitochondrial autophagy and mitochondrial membrane depolarization in SC-β cells. Shown are fold changes of genes in the indicated gene sets in day 14 SC-β cells.

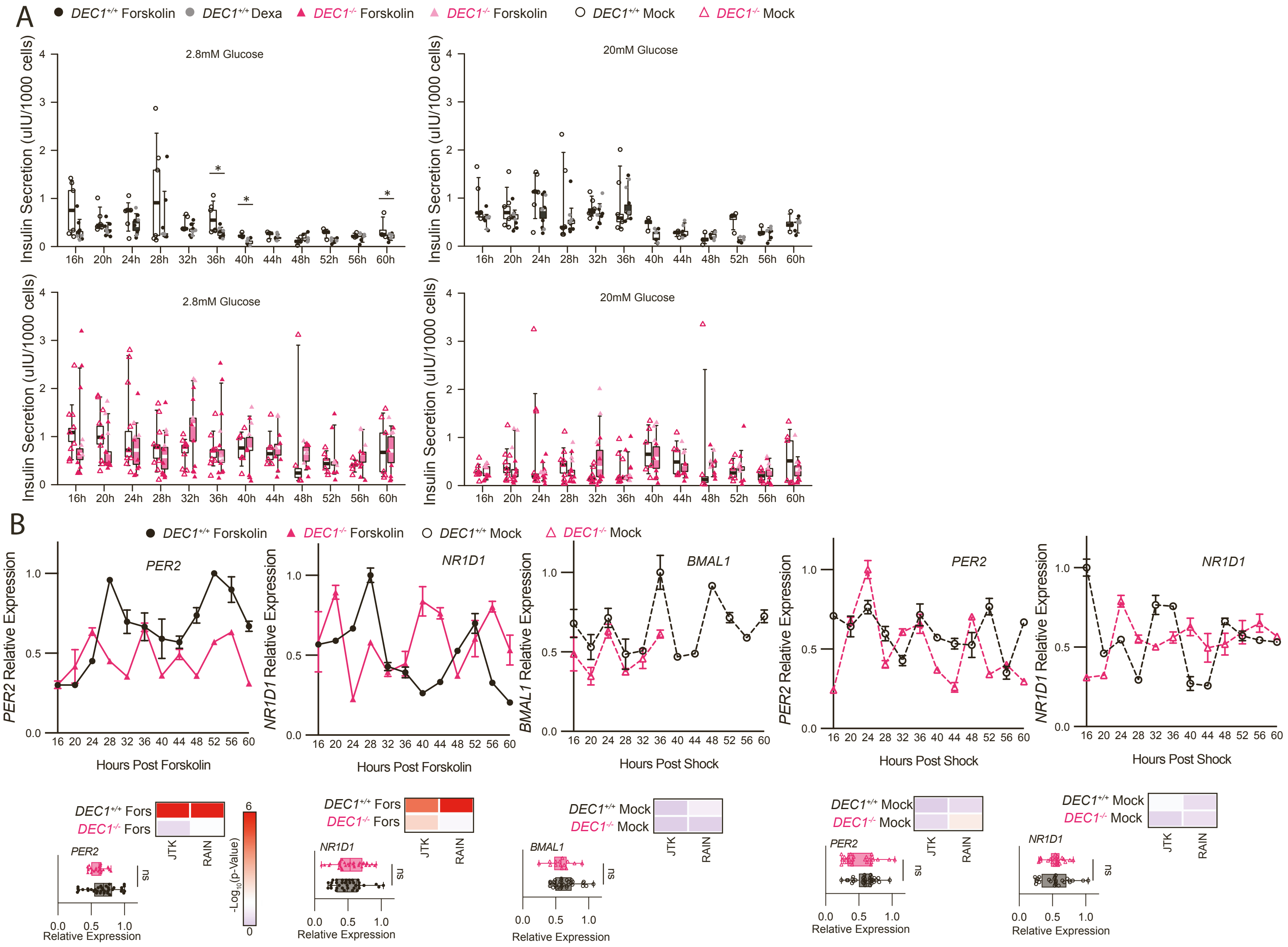

A
DEC1+/+ Forskolin
DEC1+/+ Dexa
DEC1-/- Forskolin
DEC1-/- Forskolin
DEC1+/+ Mock
DEC1-/- Mock
2.8mM Glucose
20mM Glucose
Insulin Secretion (uIU/1000 cells)
16h 20h 24h 28h 32h 36h 40h 44h 48h 52h 56h 60h
B
PER2
NR1D1
BMAL1
PER2 Relative Expression
NR1D1 Relative Expression
BMAL1 Relative Expression
Hours Post Forskolin
Hours Post Shock
DEC1+/+ Fors
DEC1-/- Fors
DEC1+/+ Mock
DEC1-/- Mock
JTK
RAIN
-Log10(p-Value)
ns
Relative Expression

Figure S3. Circadian insulin secretion and clock gene expression in *DEC1*$^{+/+}$ and *DEC1*$^{-/-}$ SC-islets. Related to Figure 3.

(A) GSIS responses show reduced insulin secretion in non-stimulatory (2.8mM) glucose at 36, 40, and 60 hours post forskolin or dexamethasone treatment in *DEC1*$^{+/+}$ SC-islets (top). Insulin secretion in 20mM glucose is shown to the right. Data are mean ± SEM. *p <0.05, unpaired Welch's t-test. N=3 differentiations, with n=50-100 SC-islets assayed from each at least in duplicate.

(B) *DEC1* loss disrupts circadian synchronization of the indicated genes. Data is expression relative to the maximum expression across the conditions shown in each panel (N = 3 differentiations with n =2 replicate measurements each). Rhythmicity p-values by JTK and RAIN analysis.

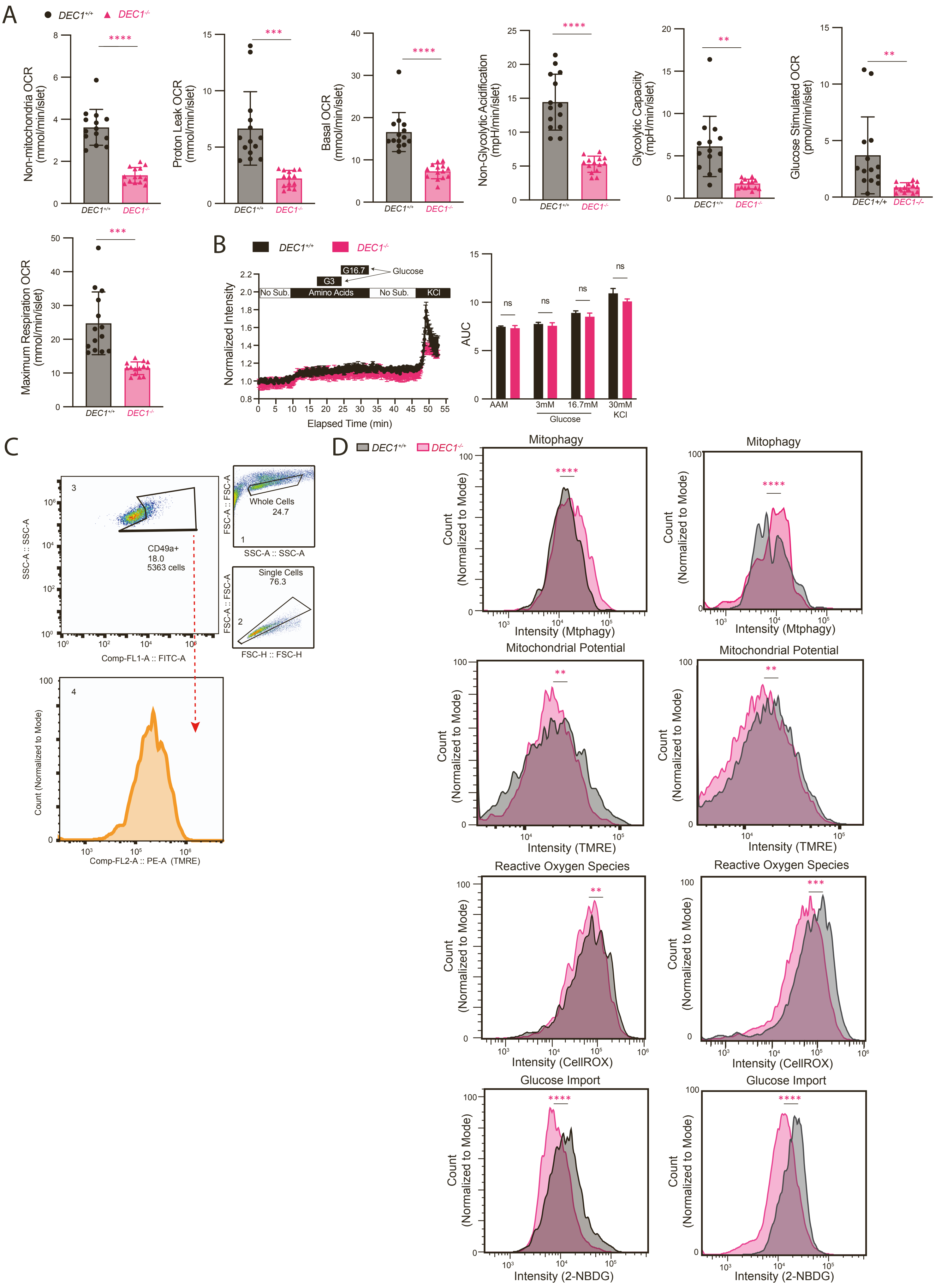
A
DEC1+/+
DEC1-/-
Non-mitochondria OCR (mmol/min/islet)
****
Proton Leak OCR (mmol/min/islet)
***
Basal OCR (mmol/min/islet)
****
Non-Glycolytic Acidification (mpH/min/islet)
****
Glycolytic Capacity (mpH/min/islet)
**
Glucose Stimulated OCR (pmol/min/islet)
**
DEC1+/+
DEC1-/-
Maximum Respiration OCR (mmol/min/islet)
***
B
DEC1+/+
DEC1-/-
G16.7
Glucose
G3
No Sub.
Amino Acids
No Sub.
KCl
Normalized Intensity
Elapsed Time (min)
AUC
ns
ns
ns
ns
AAM
3mM
16.7mM
Glucose
30mM
KCl
C
3
SSC-A :: SSC-A
CD49a+
18.0
5363 cells
Comp-FL1-A :: FITC-A
FSC-A :: FSC-A
Whole Cells
24.7
1
SSC-A :: SSC-A
Single Cells
76.3
FSC-A :: FSC-A
2
FSC-H :: FSC-H
4
Count (Normalized to Mode)
Comp-FL2-A :: PE-A (TMRE)
D
DEC1+/+
DEC1-/-
Mitophagy
****
Count (Normalized to Mode)
Intensity (Mtphagy)
Mitophagy
****
Count (Normalized to Mode)
Intensity (Mtphagy)
Mitochondrial Potential
**
Count (Normalized to Mode)
Intensity (TMRE)
Mitochondrial Potential
**
Count (Normalized to Mode)
Intensity (TMRE)
Reactive Oxygen Species
**
Count (Normalized to Mode)
Intensity (CellROX)
Reactive Oxygen Species
***
Count (Normalized to Mode)
Intensity (CellROX)
Glucose Import
****
Count (Normalized to Mode)
Intensity (2-NBDG)
Glucose Import
****
Count (Normalized to Mode)
Intensity (2-NBDG)

Figure S4. **DEC1 regulates glycolytic and oxidative metabolism in maturing SC-β cells. Related to Figure 4.**

(A) Decreases in the indicated features determined by oxygen consumption rate (OCR) and extracellular acidification rate (ECAR) analysis of $DEC1^{-/-}$ SC-islets. Data from Figure 4B experiments (N = 4 differentiations, each with n = 3-4 replicate measurements of 8 similar-sized SC-islets).

(B) Ca^{2+} influx measured by Fura-2 staining in response to no substrate (No Sub.); a physiological mixture of amino acids (AAM); AAM supplemented with 3mM glucose (G3); AAM supplemented with 16.7mM glucose (G16.7); no glucose; and 30 mM KCl, with respective AUCs quantified to the right. N=1 differentiation, n = 3 replicates with 8-12 similar-sized day 21 SC-islets. Data are mean ± SEM. *p <0.05, **p <1e-2, unpaired t-test.

(C) CD49a staining allows for SC-β cell-specific staining of mitochondria integrity stains (TMRE, CellROX, and Mtphagy) and glucose analog reporter 2-NBDG. n = 5363 cells from day 14 SC-islets.

(D) Related to Figure 4A. N=2 additional differentiations demonstrating *DEC1*-deficient day 14 SC-β cells exhibit mitochondria with increased autophagy and reduced membrane potential, along with lower reactive oxygen species generation and glucose import. n ≥ 2000 cells

Data are mean ± SEM. *p <0.05, **p <1e-2, ***p <1e-3, ****p <1e-4, unpaired Welch's t test [(A) and (B)] and Wilcoxon test (D).
